# Neutrophil extracellular traps-related lncRNAs prognostic signature for gastric cancer and immune infiltration: potential biomarkers for predicting overall survival and clinical therapy

**DOI:** 10.1007/s12672-024-01164-0

**Published:** 2024-07-19

**Authors:** Shuhan Yang, Jiahui Liang, Xin Wang, Yijun Qi, Shixin Chan, Yonghu Song, Xiaohan Pei, Zhiyao Ren

**Affiliations:** 1 Department of General Surgery, The First Affiliated Hospital of Anhui Medical University, 218 JiXi Avenue, Hefei, 230022 Anhui People’s Republic of China; 2 Faculty of Medicine and Health Sciences, Ghent University, 9000 Ghent, Belgium; 3 Department of Pathology, Division of Life Sciences and Medicine, The First Affiliated Hospital of USTC, University of Science and Technology of China, Hefei, 230036 Anhui China; 4 Intelligent Pathology Institute, Division of Life Sciences and Medicine, University of Science and Technology of China, Hefei, 230036 Anhui China

**Keywords:** Gastric cancer, Neutrophil extracellular traps, LncRNA, Prognostic model, Bioinformatics

## Abstract

Gastric cancer (GC) is one of the most common digestive tract malignant tumors in the world. At the time of initial diagnosis, it frequently presents with local or distant metastasis, contributing to poor prognosis in patients. Neutrophil extracellular traps (NETs) constitute a mechanism employed by neutrophils that is intricately associated with tumor progression, prognosis, and response to immunotherapy and chemotherapy. Despite this, the specific involvement of NETs-related long non-coding RNAs (lncRNAs) in gastric cancer remains unclear. A prognostic model for NETs-related lncRNAs was constructed through correlation analysis, COX regression analysis, and least absolute shrinkage and selection operator regression (LASSO) analysis. The predictive performance of the model was assessed using Kaplan–Meier survival curves, receiver operating characteristic (ROC) curves, facilitating the exploration of the relationship between disease onset and prognosis in gastric cancer. Additionally, differences in the tumor microenvironment and response to immunotherapy among gastric cancer patients across high- and low-risk groups were analyzed. Furthermore, a prognostic nomogram integrating the risk score with relevant clinicopathological parameters was developed. The prognostic prediction model for gastric cancer, derived from NETs-related lncRNAs in this study, demonstrates robust prognostic capabilities, serving as a valuable adjunct to traditional tumor staging. This model holds promise in offering novel guidelines for the precise treatment of gastric cancer, thereby potentially improving patient outcomes.

**Supplementary Information:**

The online version contains supplementary material available at 10.1007/s12672-024-01164-0.

## Introduction

Gastric cancer (GC) stands as one of the predominant malignancies globally, leading significantly in morbidity and mortality among the top five malignant neoplasms worldwide. According to the latest speculation released by GLOBOCAN, in 2020, the number of people suffering from gastric cancer worldwide will reach 1.089 million, and 769,000 people will die of gastric cancer, ranking fourth among all cancer types, second only to lung cancer, colorectal cancer and liver cancer [1, 2]. The insidious onset and rapid progression of gastric cancer often result in diagnoses at advanced stages in most patients [3]. Despite the implementation of perioperative management and adjuvant therapies, such as chemotherapy and radiotherapy, the 5-year survival rate remains notably compromised [4]. This dismal prognosis is primarily due to the tumor's high aggressiveness and resistance to treatment modalities [5]. Therefore, it is of great clinical significance to explore the mechanism of GC occurrence and development.

NETs are fibrous networks consisting of nuclei and granules that extend from activated neutrophil membranes [6]. The pioneering work by Demers et al. in 2012 marked the first documentation of tumor-induced NETs formation [7]. Besides their established role in inflammation and autoimmune disorders, NETs have emerged as pivotal players in various malignancies, including breast cancer, insulinoma, and lung cancer, colorectal cancer, especially gastric cancer [8–10]. Subsequently, Khan et al. confirmed the relationship between the release of NETs from neutrophils stimulated in vitro and poor prognosis in colorectal cancer [11]. Zhang et al. found that the increase of Net in peripheral blood of GC patients is related to the poor prognosis of gastric cancer patients, such as lymph node metastasis and tumor progression, and they can be used as independent prognostic factors of GC patients [12]. Additionally, Zhu et al.'s research illustrated that NETs enhance the migratory and invasive capabilities of gastric cancer cells [13].

Long noncoding RNA (lncRNA) refers to noncoding transcripts with a length more than 200 nucleotides [14]. These RNA molecules, transcribed from the genome, play crucial roles in the regulation of protein-coding genes and other non-coding RNA families [15]. Aberrant expression of lncRNAs is widely observed in various tumors and contributes significantly to oncogenic processes, they are instrumental in regulating tumor metastasis and hold potential as therapeutic targets in oncology [16–18]. Furthermore, lncRNA have highly tissue-specific expression [19], and is closely related to the occurrence and development and prognosis of various tumors, including gastric cancer [20]. At present, the therapy, prognosis, and immune microenvironment of NETS-related lncRNA in patients with non-small cell lung cancer [21], breast cancer [22], hepatocellular carcinoma [23], lung adenocarcinoma [24], and soft tissue sarcoma [25] have been studied. However, the interaction between gastric cancer and NETs-related lncRNAs is still unclear, so it is of great innovation value to study and explore them.

The purpose of this study was to explore the relationship between NETs-related lncRNAs and the prognosis, tumor immune microenvironment, immune checkpoint expression, and clinical therapy effect in gastric cancer patients. Our results provide a new strategy for predicting prognosis and designing the effective treatment of gastric cancer.

## Materials and methods

### Data acquisition

**Fig. 1:**
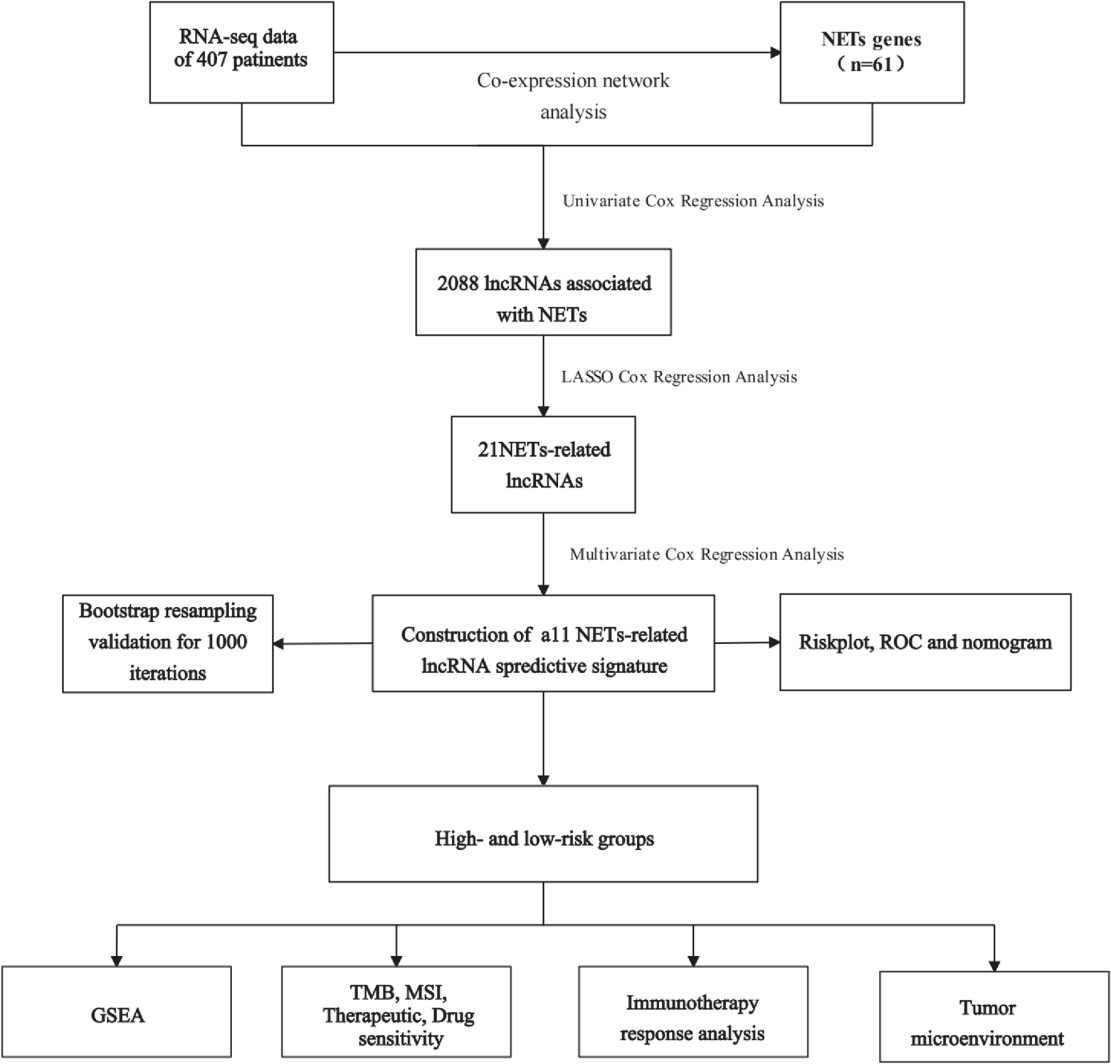
Workflow diagram

The transcriptomic data of gastric cancer, comprising 375 gastric adenocarcinoma samples, was obtained from The Cancer Genome Atlas (TCGA) database. Concurrently, relevant clinical information was also extracted from the TCGA database, including overall survival (OS) time, Progress free survival (PFS) time, age, gender, grade, and TNM stage. For the differentiation of lncRNA from messenger RNA (mRNA), we utilized a gene transfer format (GTF) file sourced from Ensemble (http://asia.ensembl.org) [26]. Furthermore, an exhaustive list of 69 NETs-related genes was meticulously collated from various scholarly publications [27] (Supplementary Table S1). The work flow of the current study is shown in Fig. 1.

### Identification of NETs-related lncRNAs and construction of prognostic signature

We utilized the Strawberry Perl software (https://strawberryperl.com/) to distinguish the downloaded transcription data into lncRNAs and mRNAs. Subsequently, co-expression analysis of 69 NETs genes was conducted to determine the expression level of lncRNAs related to NETs. The limma R package was employed to extract the NETs gene expression matrix. The Pearson correlation coefficient (|COR|≥ 0.4, p < 0.001) served as the criterion for identifying NETs-related lncRNAs (Supplementary Table S2). To illustrate the co-expression relationship between NETs genes and NETs-related lncRNAs, we employed the dplyr, ggplot2, and ggalluvial R packages to generate a Sankey diagram. For prognostic prediction involving NETs-related lncRNAs, we initially identified lncRNAs significantly correlated with gastric cancer patient prognosis through univariate Cox regression analysis (P < 0.05). Subsequently, least absolute shrinkage and selection operator (LASSO) regression analysis refined our screening process, enhancing the model's precision. The final step involved constructing a multivariate Cox regression prognostic model for NETs-related lncRNAs, integrating it with the minimum Akaike Information Criterion (AIC) value. This was further visualized in a forest plot to elucidate the the Hazard Ratio (HR) of these lncRNAs. The model's efficacy was internally validated through a 1000 times bootstrap method [28]. We calculated the mean and standard deviation of time-dependent area under the curve (time-AUC) and the concordance index (C-index) using the Surv, Intt, glmnet, Survminer, Time ROC, Caret, Mtcars, and pheatmap R packages.

The risk score for gastric cancer patients was computed using the following formula: Risk Score = ∑Coef(K) × N(K), where Coef(K) and N(K) respectively denote the regression coefficient of NETs-related lncRNAs and the expression level of lncRNAs. Subsequently, we utilized the corrplot R package to analyze the relationship among the 11 NET-related lncRNAs involved in model construction and generated a visual heatmap. Furthermore, the optimal cutoff value was determined using Surv_cutpoint from the SurvMiner R package, effectively stratifying patients into low- and high-risk groups based on maximal survival differences.

### Validation of prognostic signature

To assess the predictive efficacy of the prognostic signature, we conducted a log-rank test comparing OS and PFS across varied risk groups. Kaplan–Meier survival curves were generated for this purpose by using ggsurvlot R package. Additionally, Kaplan–Meier survival curves were employed to examine the correlation between clinicopathological features and OS in distinct risk groups, providing further insight into the prognostic signature’s value. The risk curve, survival state diagram and risk heat map were constructed by pheatmap R package, so that the influence of NETs-related lncRNAs on prognosis could be comprehensively analyzed. Independent prognostic analysis further substantiated the model’s predictive capacity. Additionally, we calculated and illustrated the concordance index (C-index) for independent prognostic factors, confirming our model’s predictive validity. In order to evaluate the predictive ability of the prognosis model in predicting the 1-,3-and 5-year survival rates, Survival, Surv Miner and timeROC R Packages were used to draw the Receiver operating characteristic (ROC) curve and calculate the area under the curve. The ComplexHeatmap R package was utilized to construct a detailed heatmap, highlighting the relationship between various clinicopathological parameters and risk stratification.

Based on the risk score, age and TNM stage, we used regplot, survival, and rms R package to establish the nomogram of OS for 1, 3 and 5 years. The calibration curve and decision curve analysis (DCA) were constructed to predict the accuracy of clinical prognosis nomogram.

### Enrichment analysis

We identified the differentially expressed mRNAs between the high- and low-risk groups using the limma, pheatmap, and ggplot2 R packages. The filtering criteria were set as | log2fold change (FC) |> 1 and false discovery rate (FDR) < 0.05. We visualized the results using visual heatmaps and volcano plots, focusing on the top 50 differentially expressed genes. To further elucidate the characteristics of the 11 NETs-related lncRNAs within the prognostic signature, we utilized the tidyverse, tibble, data.table, magrittr, and survival R packages to construct correlation heatmaps with the differentially expressed mRNAs. We taked the correlation coefficient |COR|> 0.4, p < 0.001 as the standard of differentially expressed genes co-expression with NETs related lncRNAs. Additionally, we visualized the co-expression patterns using Sankey diagrams. The above analyses were performed using the dplyr, ggplot2, ggalluvial, Limma, and pheatmap R packages. To explore the difference of biological pathways of two risk groups, to explore the function, biological process, and to clarify the biological pathways different risk groups, we employed the GSEA (Gene Set Enrichment Analysis) method to analyze GO (Gene Ontology), KEGG (Kyoto Encyclopedia of Genes and Genomes), and Hallmark gene sets. The top five significant pathways for each analysis were presented.

### Tumor Mutation Burden (TMB), Microsatellite instability (MSI), Cancer stem cell (CSC) and prognostic signature

The Tumor Mutation Burden (TMB) represents the number of mutations per million bases. We computed the mutation frequency and total mutation count for all samples, subsequently stratifying them into high TMB and low TMB groups. We then analyzed the differences in survival rates between these groups based on patient survival information. Comparisons of gene mutation statuses between the high- and low-risk groups were conducted, and waterfall plots were generated to visualize the results. Additionally, TMB variances and correlations were analyzed for both risk groups. Furthermore, we examined the relationships between the risk score and Microsatellite Instability (MSI) and Cancer Stem Cell (CSC) statuses.

### Tumor micro-environment (TME), immune cell infiltration, checkpoint, and immunotherapy

We employed the CIBERSORT method to analyze the content of various immune cell types in all samples, visualizing the results using the pheatmap and corrplot R software packages. The Estimate and limma R packages were utilized to determine the proportions of immune cells and stromal cells in each sample, facilitating comparisons of cell infiltration abundance between the high- and low-risk groups. Additionally, we analyzed the differences in Stromal score, Immune score, and Estimate score between the two groups. Using TIMER, CIBERSORT, and other algorithms, we investigated the correlation between immune cells and risk scores, presenting the results in a bubble chart format. Survival analysis of immune cell infiltration was conducted using the LIMMA, Survminer, and SurvMiner R packages, with Kaplan–Meier curves drawn to visualize the findings. We employed the ssGSEA method to score immune infiltrating cells in gastric cancer samples, analyzing differences between the two risk groups and presenting them in a boxplot format. Furthermore, we utilized the Wilcox test to compare the differences in gene expression of 47 immune checkpoints between the high- and low-risk groups.

Additionally, we analyzed the expression of immune checkpoint inhibitor (ICI) related molecules in different subgroups and investigated differences in the IC50 of immunotherapy in patients with gastric cancer.

## Results

### Identification of NETs-related lncRNAs and construction of prognostic models

**Fig. 2:**
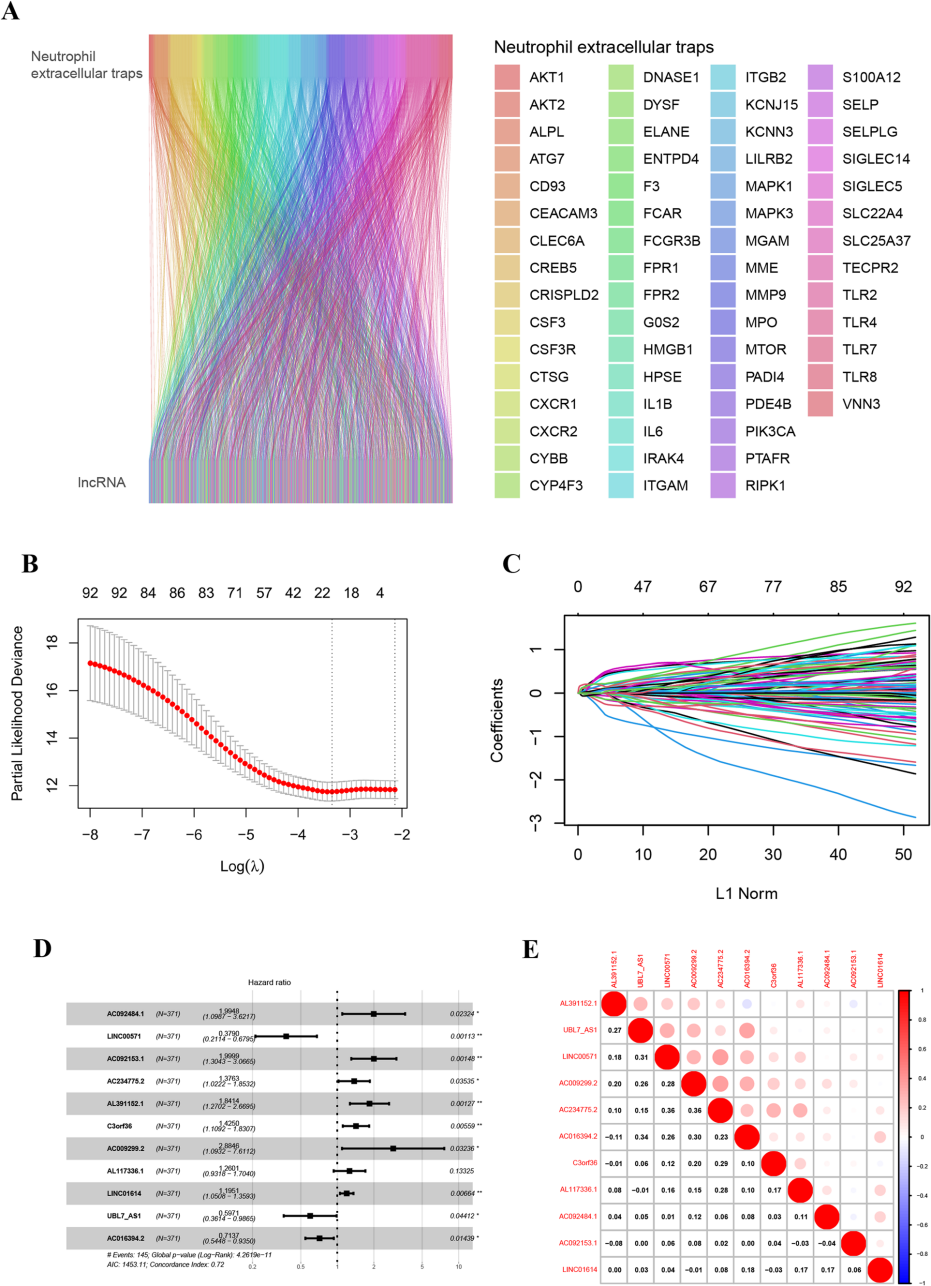
Identification of prognostic NETs-related lncRNAs. **A**Sankey diagram of NETs-related lncRNAs in gastric cancer. **B**, **C** LASSO variation trajectory of each independent variable (**B**) and LASSO coefficient profile (**C**). **D** Forest Plot for Multivariate Cox Regression Analysis. **E** Heatmap for the correlation between 11 NETs-related lncRNAs

We curated a total of 69 genes associated with NETs and employed a criterion for identifying NETs-related lncRNAs, utilizing a Pearson correlation coefficient |COR|≥ 0.4 and p < 0.001. A Sankey map (Fig. 2A) and Table S1 illustrated the corresponding relationship between the 61 NETs genes and 2088 NETs-related lncRNAs. Univariate COX regression analysis showed that 94 NETs-related lncRNAs could affect the prognosis (Supplementary Table S3). Using the LASSO regression algorithm and bootstrap, 21 Nets-related LncRNAs were identified based on the minimum partial likelihood of the best λ value and deviation (Fig. 2B, C, Supplementary Table S4). Multivariate Cox regression analysis was performed on these 21 LncRNAs, and a risk model consisting of eleven LncRNAs was obtained (Fig. 2D). The c-index is 0.72. The molecular formula of the model was as follows:

Risk Score(RS) = (0.370944109* AC092484.1–0.359529802* LINC00571 + 0.382430253* AC092153.1 + 0.054296625* AC234775.2 + 0.307149302* AL391152.1 + 0.170951498* C3orf36 + 0.419843652* AC009299.2 + 0.06115273* AL117336.1 + 0.083358899* LINC01614 -0.196147433* UBL7_AS1 -0.112310037* AC016394.2). To explore the expression correlation between the 11 NETs-related LncRNAs, a correlation heatmap was generated based on TCGA samples (Fig. 2E).

### Validation of prognostic NETs-related lncRNA signature

**Fig. 3:**
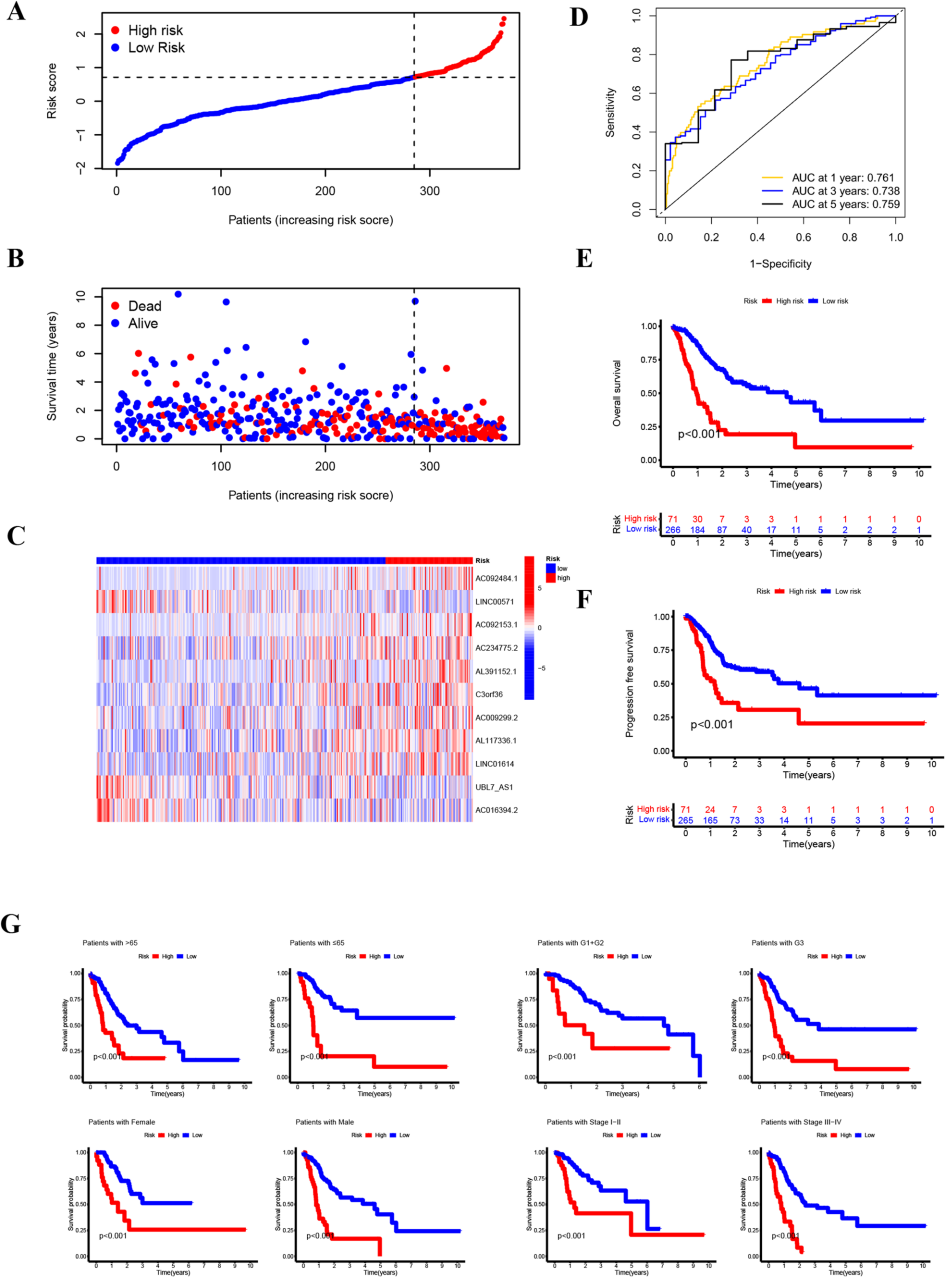
Validation of prognostic NETs-related lncRNA signature. **A**–**C** Ranked dot, heat map and scatter plots of the model gene expressions in TCGA datasets. **D** ROC curves and AUCs for 1-, 3-, and 5-year survival rates. **E** K-M survival curve of overall survival of patients in high- and low-risk groups **F** K-M survival curve of progress free survival of patients in high- and low-risk groups **G** K-M survival curve of patients with different pathological characteristics in high- and low-risk groups

The signature was evaluated for its predictive capability through a risk plot and KM survival analyses. The R ‘heatmap’ was utilized to map the risk plot. Patients were categorized into high- and low-risk groups based on the optimal cutpoint identified by the survminer package (Fig. 3A). Remarkably, with an increase in RS, there was a gradual rise in the proportion of deceased patients, coupled with a progressive decrease in survival duration (Fig. 3B).

Figure 3C illustrates significant variations in the expression levels of 11 lncRNAs (AC092484.1, LINC00571, AC092153.1, AC234775.2, AL391152.1, C3orf36, AC009299.2, AL117336.1, LINC01614 with higher expression in the high-risk group, and UBL7_AS1, and AC016394.2 with higher expression in the low-risk group). As shown in our set, AUC at 1 year was 0.761, AUC at 3 years was 0.738, and AUC at 5 years was 0.759 (Fig. 3D). After conducting 1000 times of Bootstrap resampling with replacement, the performance metrics of the risk scoring model demonstrate relative stability across different samples (Supplementary Table S5). At 1 year, 3 years, and 5 years, the average AUC values are 0.7449, 0.7232, and 0.7393, respectively, with standard deviations of 0.0114, 0.0154, and 0.0261. The mean c-index is 0.7260, with a standard deviation of 0.0219.

Validation of the Kaplan–Meier survival analysis through patient stratification into high and low groups demonstrated significant disparities in survival status (P < 0.001), with notable distinctions also observed in progression-free survival (Fig. 3E, F). Subsequently, we re-stratified patients based on their clinical information and further validated the model. We compared the OS rates between high- and low-risk groups among patients grouped by age (above and below 65 years), tumor stage (I-II and III-IV), gender (male and female), and tumor grade (G1 + G2 and G3). We further confirmed that the risk score (RS) model performed effectively in different clinical scenarios (Fig. 3G). The clinical heatmap showed the differences in clinical characteristics between high and low-risk groups of patients (Supplementary figure S1).

### Evaluation of the RS model

**Fig. 4:**
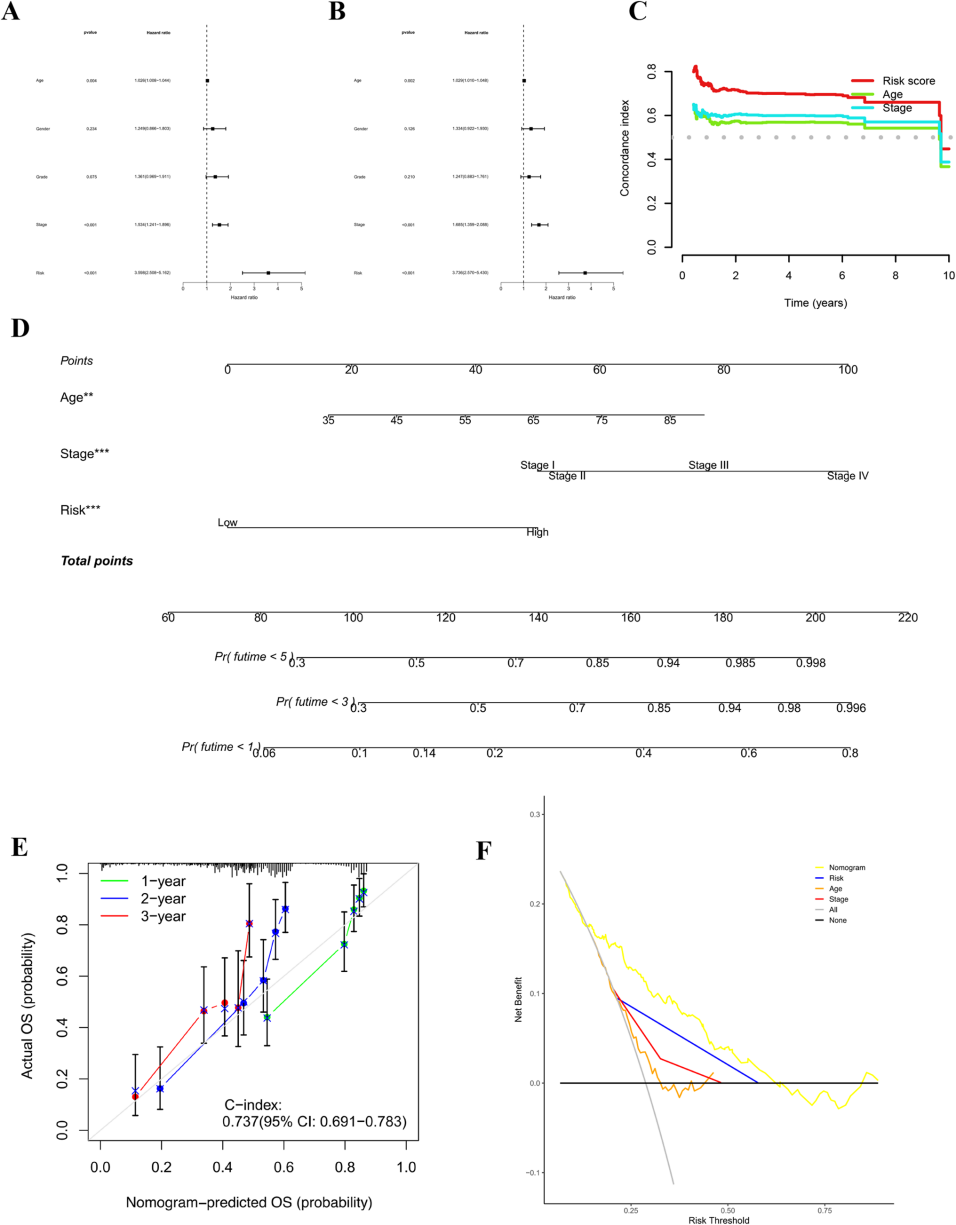
Independent prognostic value of NETs-related lncRNA signature and nomogram. **A** Univariate Cox Regression Analysis. **B** Multivariate Cox Regression Analysis. **C** C-index. **D** The nomogram used to calculate the survival rates of 1-, 3-, and 5-years for patients with GC. **E** Calibration curves of the nomogram. **F** DCA curve of the risk model

Afterward, univariate and multivariate Cox regression analyses were conducted to assess whether the prognostic characteristics of the signature remained independent of gender, age, tumor grade, and tumor stage (Fig. 4A, B). The resulting data exhibited that the established signature remained an independent prognosis-predictive factor.

Moreover, compared to two other multifactor independent prognostic factors (Age, Stage), the concordance index of the risk signature suggested that the signature could serve as a more reliable reference index in clinical settings (Fig. 4C).

Patients were randomly selected for scoring, total point = point (Age) + point (Stage) + point (RS) below by combining RS and clinicopathological features, using the nomogram (a quantitative method), as shown in Fig. 4D. The total score corresponds to the scale in the figure and is utilized to predict patient survival. The Decision Curve Analysis and Calibration Curve collectively corroborated the accuracy and stability of the model, providing comprehensive validation for its performance (Fig. 4E, F).

### Differential mRNA analysis and functional enrichment analysis between high-risk and low-risk groups

**Fig. 5:**
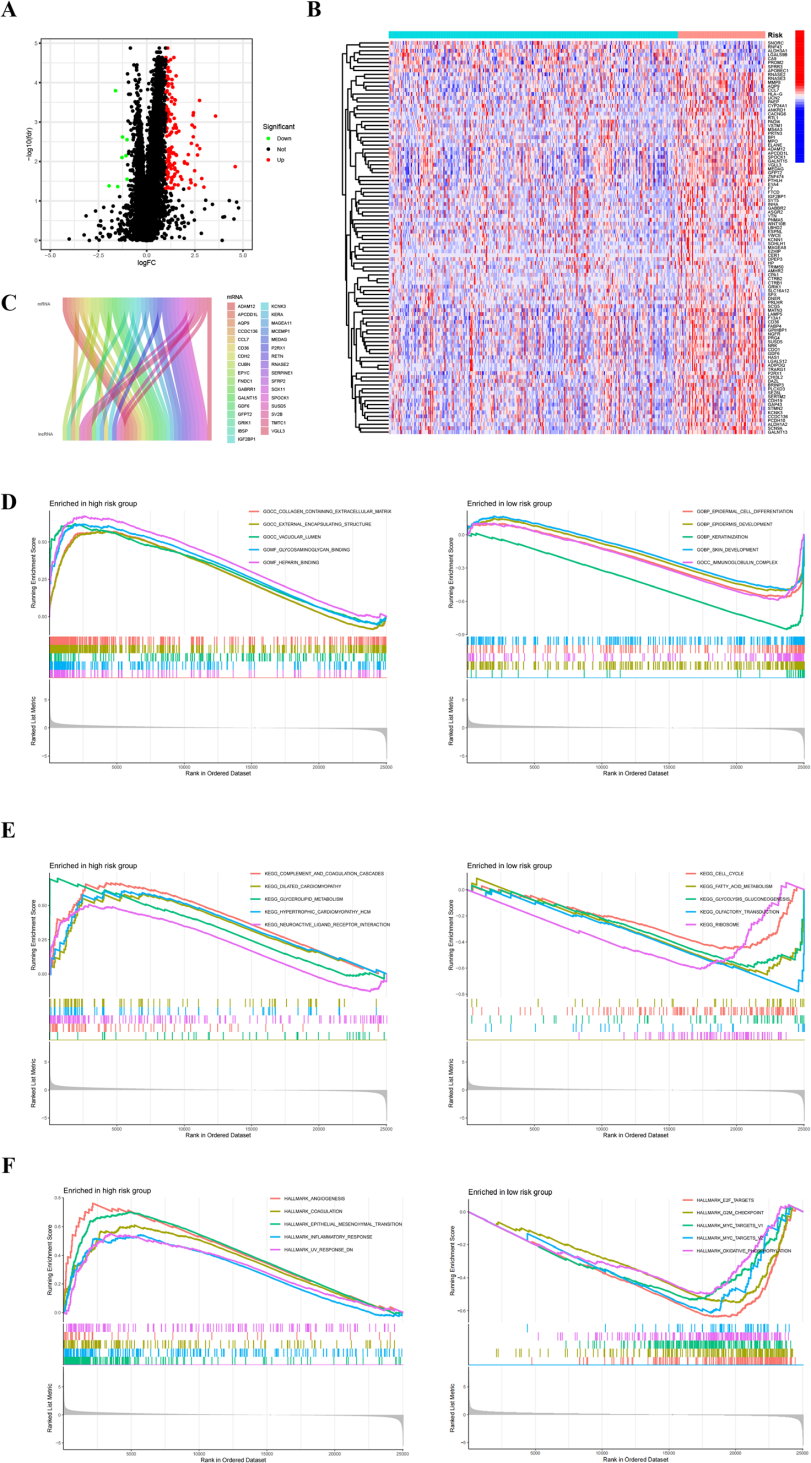
Differential mRNA analysis and GSEA between high- and low-risk groups. **A** Volcano plot of the top 50 differentially expressed genes between high- and low-risk groups. **B** heatmap of differential mRNA expression between high- and low-risk groups. **C** Sankey diagram for differentially expressed genes co-expressed with 11 NETs-related lncRNAs. **D** GSEA analysis based on GO gene set of high- and low-risk groups. **E** GSEA analysis based on KEGG gene set of high- and low-risk groups. **F** GSEA analysis based on hallmarker gene set of high- and low-risk groups

Through screening between the high- and low-risk groups, with criteria of |log2 fold change (FC)|> 1 and false discovery rate (FDR) < 0.05, we successfully identified 159 differentially expressed mRNAs in the high- and low-risk groups. (Supplementary Table S7). Figure 5A presented the differentially expressed genes. Figure 5B depicts the expression patterns of 159 differentially expressed mRNAs between high- and low-risk groups. By utilizing a correlation coefficient threshold of |COR|> 0.4 and p < 0.01 as criteria for co-expression with NETs-related lncRNAs, a total of 33 co-expressed mRNAs were identified. A Sankey map (Fig. 5C) illustrated the corresponding relationship between NETs-related lncRNAs and 33 differentially expressed mRNAs. A heatmap (Supplementary figure S2) illustrated the the correlation between 11 NETs-related lncRNAs and differential mRNA expression between high-risk group and low-risk group. GSEA analysis was conducted for a detailed assessment of the biological functions between the high- and low-risk groups. The results indicated that the top five enriched pathways in the two risk groups were distinct from each other (Fig. 5D–F, Supplementary Table S8).

### Different immune landscapes in the two risk groups

**Fig. 6:**
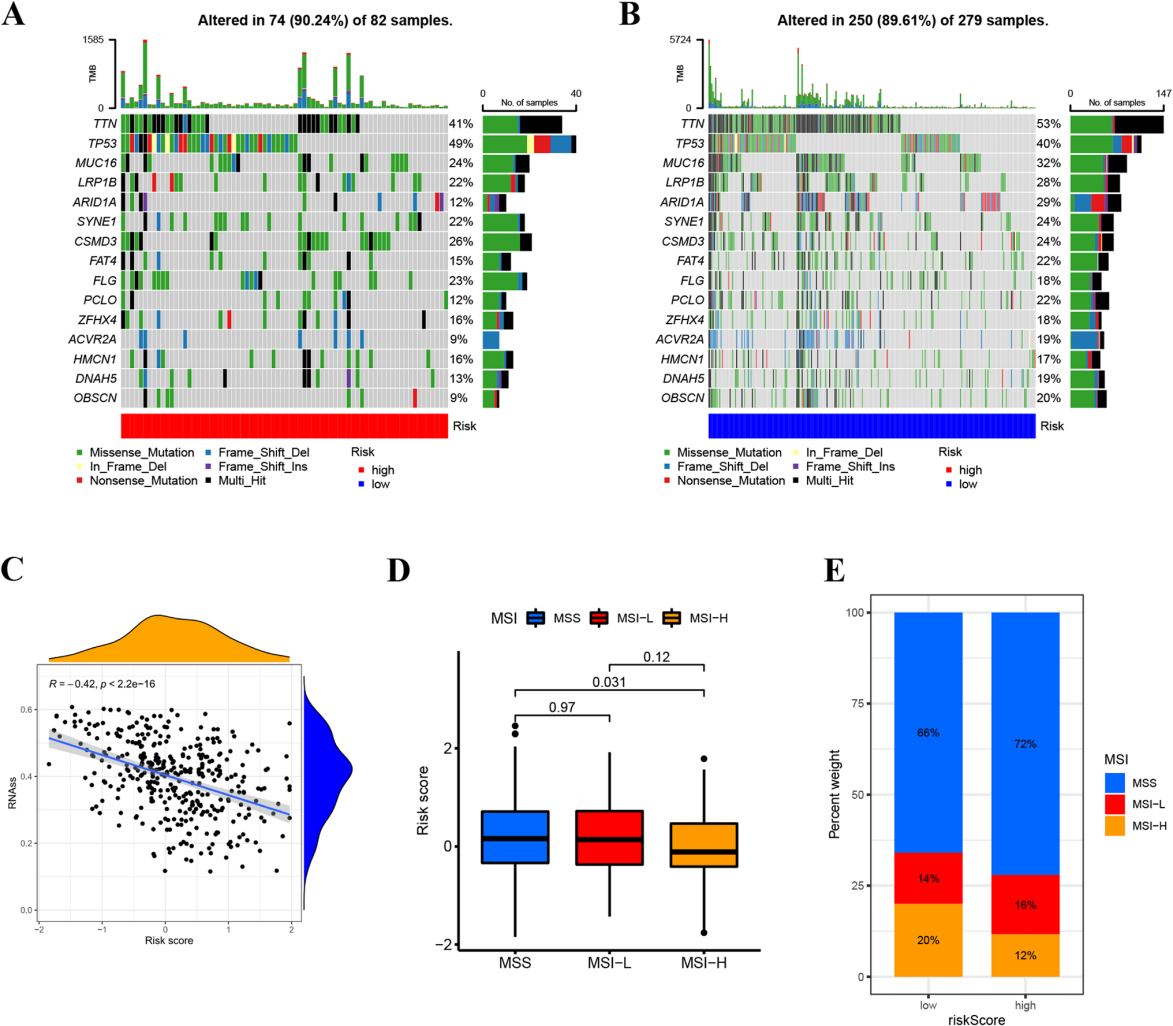
Mutation, CSC, MSI analysis of the prognostic signature. **A**, **B** The somatic mutation features waterfall plot determined by high- and low-risk scores. One patient was represented by each column. The correct number represented each gene's frequency of mutation, and the upper barplot displayed TMB. The proportion of each variant type was displayed in the right barplot. **C** Association between the CSC index and the risk score. **D**, **E** Relationships between MSI and risk score

The mutation rate of the high-risk group was 90.24% (Fig. 6A). The mutation rate of the low-risk group was 89.61% (Fig. 6B). The waterfall diagram shows that the mutation genes in the high- and low-risk groups are mainly TNN, TP53, MUC16, LRP1B, ARID1A, and the mutation rates of these genes are different in the high- and low-risk groups. TNN had the highest mutation frequency in the low-risk samples, while TP53 had the highest in the high-risk groups. The mutation probability of TP53 and TNN in the high-risk group were 49% and 41%, while the mutation frequency of TP53 and TNN in the low-risk group were 40% and 53%. In Supplementary figure S3, we analyze and demonstrate the predictive value of mutation state combined with clinical prediction model for patients' prognosis. There was a negative correlation between RS and stem cells. The higher the RS, the lower the content of stem cells (p < 0.001) (Fig. 6C). Compared to MSS and MSI-L groups, patients in the MSI-H group exhibit lower RS scores (Fig. 6D). In the low-risk group, there is an inclusion of 20% MSI-H gastric cancer patients, while this proportion decreases to 12% in the high-risk group (Fig. 6E).

### Correlation between prognostic lncRNAs and tumor immunity

**Fig. 7:**
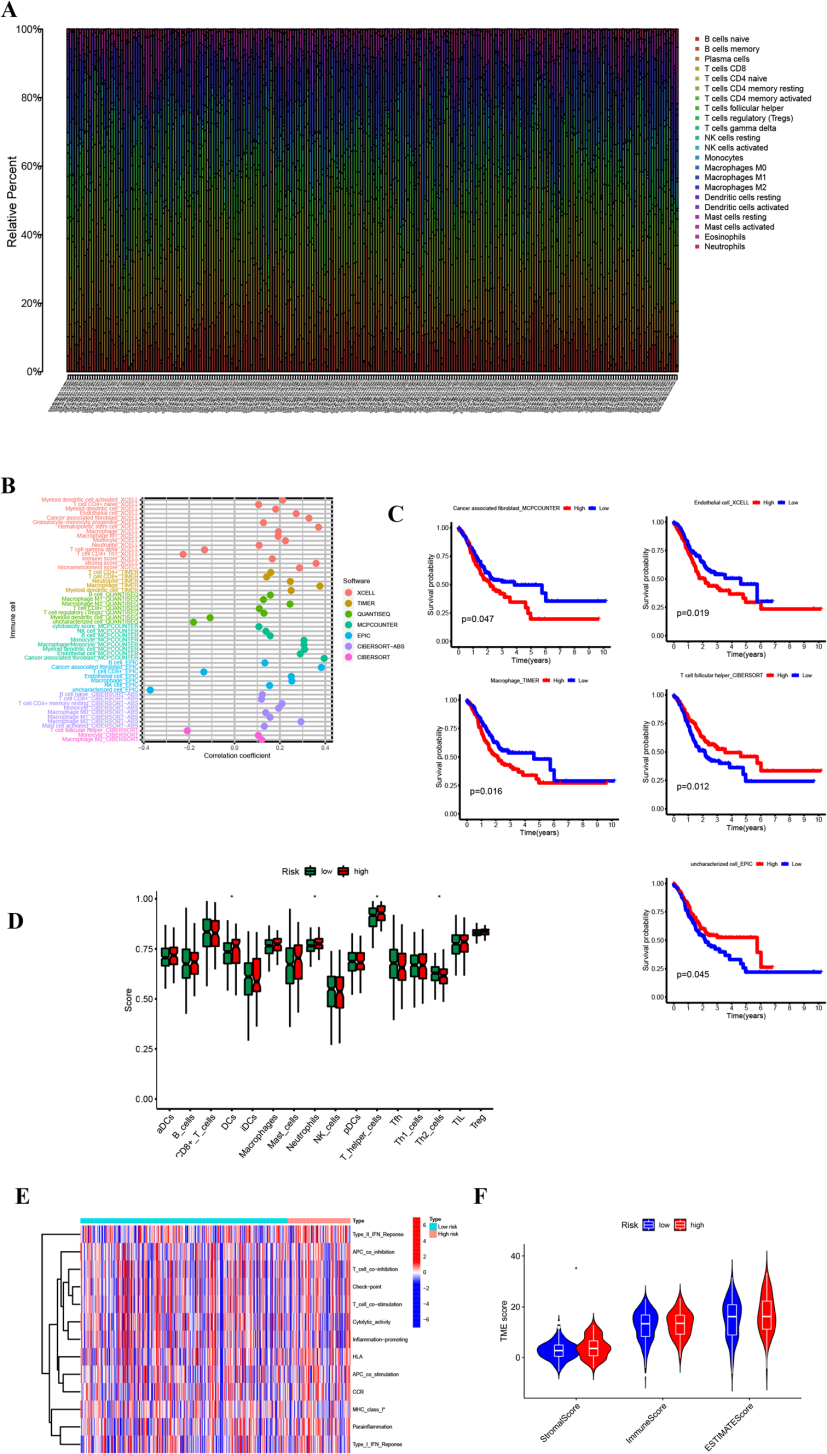
The immune infiltration landscape between high- and low-risk groups. **A** Content of immune cell types in all samples **B** Estimation of immune-infiltrating cells in GC **C** The overall survival rate of patients by risk group and immune cells was analyzed by Kaplan-Meyer curve. **D** The score of the infiltrating immune cells. **E** heatmap of immune function difference analysis **F** Estimate score of the expression profile in between high- and low-risk group

We utilized the CIBESORT package to analyze the content of various immune cell types in all samples (Fig. 7A). Subsequently, multiple algorithms, including XCELL, QUANTISEQ, TIMER, EPIC, MCPCOUNTER, CIBERSORT, and CIBERSORT-ABS (as shown in Fig. 7B), were applied to investigate fluctuations in immune infiltration between the groups. The results demonstrated that positive correlation coefficients were widespread, suggesting that patients with a higher index were in a state of immune enhancement. In various immune algorithms, there is a clear positive correlation between risk scores and cancer-associated fibroblasts. Figure 7C illustrates the effect of immune cells on patient survival.

We performed a single sample gene set enrichment analysis (ssGSEA) on high and low risk groups. From Fig. 7D, we can see DCs, neutrophils and T helper cells were highly expressed in high-risk group. Besides, Th2_cells were lowly expressed in low-risk group. We further visualized the differences in immune function between the high- and low-risk groups using a heatmap (Fig. 7E). To explore the tumor microenvironment (TME) landscape which plays an important role in progression and treatment of tumors, multiple immune assessment algorithms were applied to the high- and low-risk groups. According to the ESTIMATE algorithm (Fig. 7F), we noted that in the high-risk group, the patients’ ImmuneScore (p < 0.05) were significantly higher than those in the low-risk group.

### Drug sensitivity and immunotherapy responses in the high- and low-risk groups

**Fig. 8:**
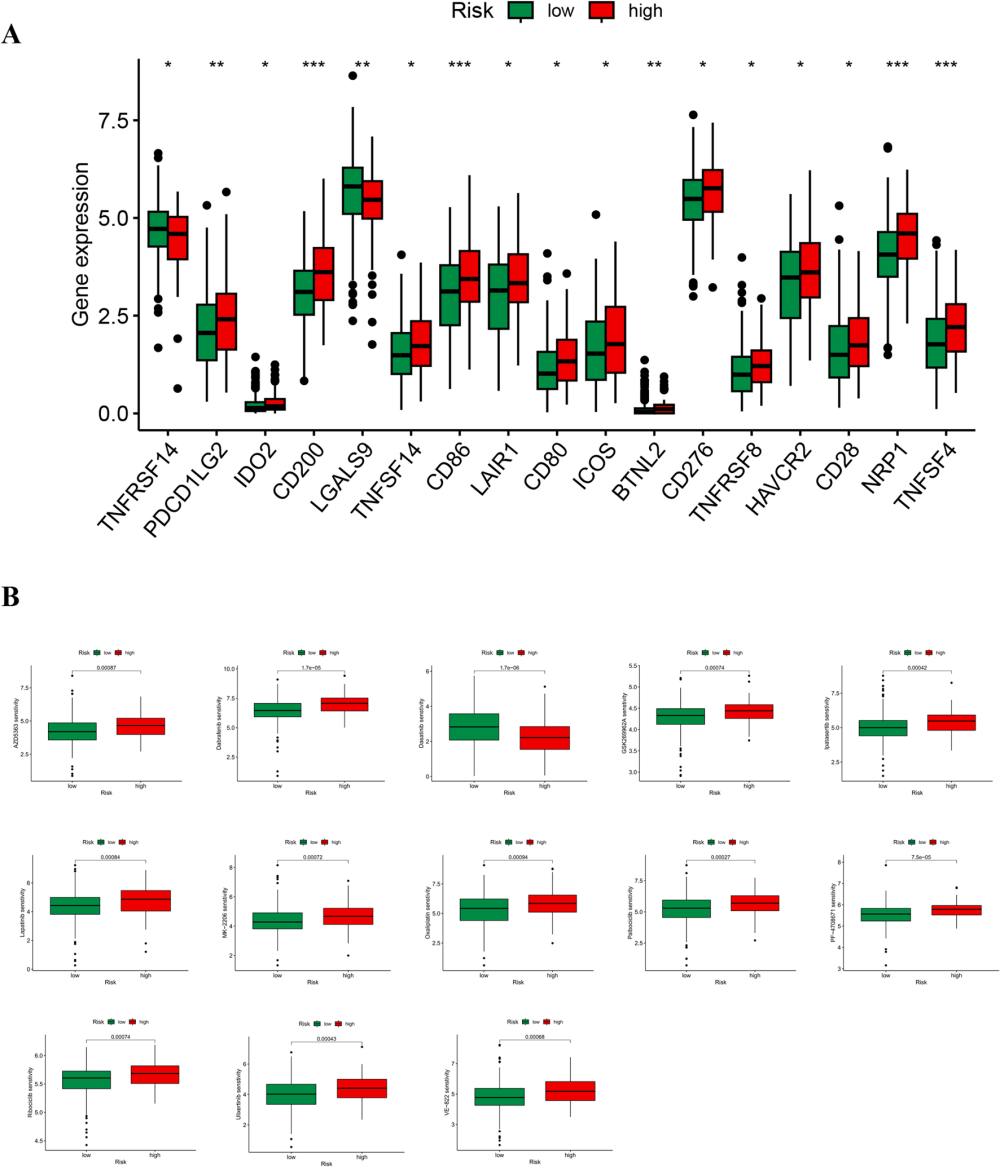
Drug sensitivity and immune checkpoints between high- and low-risk groups. **A** Differential expression analysis of the immune checkpoint genes between the high- and low-risk groups. *p < 0.05; **p < 0.01; ***p < 0.001. **B** The boxplots for the drug sensitivity analysis. IC50, the half-maximal inhibitory concentration

In order to explore the application of immune checkpoint inhibitor (ICI) in gastric cancer patients, we conducted immune checkpoint analysis. Most immune checkpoint genes were highly expressed in the high-risk group, and only TNFRSF14 and LGALS9 genes were highly expressed in the low-risk group (Fig. 8A). Drug therapy is an important part of the treatment for gastric cancer, and relevant research has always been concerned. Therefore, we calculated the IC50 value of gastric cancer drugs and explored the relationship between risk score and drug resistance. The results showed that the IC50 of dasatinib was higher in the low-risk group, the sensitivity of other targeted drugs (AZD5363, Dabrafenib, GSK269962A, Ipatasertib, Lapatinib, MK-2206, Oxaliplatin, Palbociclib, PF-4708671, Ribociclib, Ulixertinib, VE-822) in low-risk group was higher than that of the high-risk group (Fig. 8B).

## Discussion

Gastric cancer is a common tumor of digestive system, and its incidence rate has regional differences, and the incidence of young patients is gradually increasing [1]. Because there is no specific manifestation, most patients were diagnosed as advanced stage for the first time, these patients often have poor prognosis [29]. It has made great progress in the fields of surgery, chemotherapy, targeted drugs. As widely acknowledged, neutrophils stand as pivotal immune cells within the human body, executing their immune functions through a myriad of mechanisms, notably including the production of NETs [30]. The formation of NETs is pervasive across infectious diseases, autoimmune conditions, and malignancies [31–34], various studies have documented their involvement in cancer immunoediting, progression, and metastatic dissemination [35, 36]. For instance, a study delineated the ability of NETs to induce the migration, invasion, and angiogenesis of pancreatic cancer cells [37]. The burgeoning body of evidence underscores the critical role of NETs in tumor immunity, inflammation, and the tumor immune microenvironment. LncRNA is a non-coding RNA fragment [14], and some scholars have used lncRNA to construct a prediction model to predict the prognosis of gastric cancer patients [38, 39]. In the current era of precision medicine, the accurate prediction of individual outcomes stands as a cornerstone for personalized treatment strategies. Scholars have endeavored to leverage NETs-related genes to construct various tumor prognostic models, encompassing lung cancer [21], breast cancer [22], hepatocellular carcinoma [23], illustrating the close association between NETs and the prognosis of diverse malignancies. However, the utilization of NETs-related lncRNAs to forecast the prognosis of gastric cancer patients remains unexplored. Our study represents a pioneering effort in this domain, holding significant potential clinical value.

Next, we constructed and validate a suitable clinical prognosis model, which was based on the NETs risk score and the patient's clinical survival data. Univariate Cox regression analysis and Lasso regression analysis were used to screen out lncRNAs related to NETs. Then the multivariate Cox regression analysis is used to construct the model containing 11 lncRNAs, and the lasso regression algorithm is combined, which not only avoids the over-fitting of the risk model, but also improves the prediction efficiency of the prediction indicators for independent data. In addition, unlike most studies that use the median as the dichotomy standard, we adopt a result-oriented method to determine the best cutoff value, thus distinguishing different risk groups more scientifically. Thus, 11 NETs related lncRNAs were selected to participate in the construction of prognosis model. Effective internal validation is very important to improve the generality of the model [40]. Compared with other internal verification methods, we adopt Bootstrap method, which has the advantage that can use every sample for validation [41]. Therefore, the results of our internal validation confirm the reliability of the model construction process. Consequently, the model stratifies patients into high- and low-risk groups based on NETs-related lncRNAs. Kaplan–Meier survival analysis revealed significantly higher OS and PFS in the low-risk group compared to the high-risk group, demonstrating statistically significant differences. ROC curve analysis with satisfactory 1-,3-,5-years AUC demonstrated favorable prediction performance of the prognosis model, affirming its robust predictive capability.

Significant differences in survival rates between high-risk and low-risk groups were observed across specific clinicopathological features, indicating the general applicability of this model to patients with gastric cancer at different stages. For further illustrating the independent value of the model, univariate and multivariate Cox regression analyses were performed, revealing that the risk score, age, and stage were independent prognostic factors. Moreover, c-indexes were calculated, demonstrating superior evaluation efficiency of the risk score compared to the other two factors. To elucidate the efficiency of the combination of risk score, age, and tumor stage for predicting OS, we developed a nomogram incorporating these three independent prognostic factors. Moreover, DCA and Calibration Curve collectively affirmed the accuracy and stability of the model. This suggests that nomogram can be used efficiently and concisely in application and clinical scenarios.

Through the GSEA analysis using GO, KEGG and Hallmark gene sets, we noticed that the cellular components or pathways such as extracellular matrix, completion and coagulation and epithelial mesenchymal transition (EMT) were obviously enriched in the high-risk group. More and more evidence show that the changes of the composition and assembly of extracellular matrix strongly affect the function and behavior of cells. ECM and EMT are closely related to the occurrence, development and prognosis of gastric cancer [42–44]. In addition, complement and coagulation pathways were identified as an essential enhancer that include nucleotide excision repair [45]. Combined with the above, it can be inferred that NETs may mediate the poor prognosis of high-risk patients through these cell components or pathways.

Then, based on the risk score, we explored the characteristics of tumor immune microenvironment, the differences of TMB, immune score and immune checkpoint expression level between high-risk population and low-risk population. In addition, targeted drug sensitivity analysis can guide clinical practice and help patients with gastric cancer to choose more effective drug treatment. Mutations in the TP53 gene are associated with a poor prognosis in gastric cancer patients [46]. Our analysis showed that the mutation rate of TP53 gene was higher in high-risk patients. In addition, microsatellite-unstable tumors are hyper-mutated intestinal-subtype tumors, these have a better prognosis and the lower frequency of recurrence (22%) [46]. Notably, the proportion of MSI-H tumors in the low-risk group was higher, affirming the high clinical utility of our prognostic model. Tumor immune infiltrating cells constitute a crucial component of the tumor immune microenvironment, intricately linked to tumor immunotherapy sensitivity and patient prognosis [47, 48]. Employing the ssGSEA algorithm, we examined the immune status of different groups, revealing a higher abundance of neutrophils and T helper cells in the high-risk group. Neutrophils is closely related to the poor prognosis of gastric cancer patients [49], this is consistent with our research. We postulate that the poor prognosis of the high-risk group may stem from the involvement of neutrophils in the formation of NETs. Therefore, our model may also predict the extent to which tumors promote immune cell infiltration.

However, due to the heterogeneity of gastric cancer and other reasons, the clinical efficacy of drug therapy is often not satisfactory [50]. PD-1 and CTLA4 are two classic immunotherapy targets for gastric cancer at present [51]. Effective immunotherapy can delay tumor progression and prolong patients' survival [52]. Therefore, searching and selecting effective immune checkpoints has always been the focus of research. Our research shows that the high-risk group is generally significantly higher than the low-risk group in terms of immune checkpoints, and the risk scores are significantly positively correlated, which will provide important guidance for developing new immunotherapy targets. Consequently, combined with drug sensitivity analysis, most chemotherapy drugs, target drugs are more sensitive in the low-risk group. This may partly explain why low-risk patients have a better prognosis. Dasatinib is one of the representative drugs of tyrosine kinase inhibitors [53], and it is the only targeted drug identified in this analysis that is more sensitive to high-risk patients. Studies have shown that it plays a role in the treatment of gastric cancer [54], which suggests that this drug may be a potential drug for treating high-risk patients.

Our research reveals the important role of NETs related lncRNAs risk score in evaluating gene expression patterns, survival, prognosis and immune cell infiltration characteristics, and guiding tumor immunotherapy. We not only validate the validity of the model, but also our model is based on RNA expression, and its detection can be completed only by simple qPCR, which has broad clinical application prospects. Therefore, our model and its derived nomogram can become an effective tool to predict the prognosis of gastric cancer patients in clinical work after further validation. At present, we are actively applying for grant and collecting clinical samples to further validate the reliability of the model.

## Limitation

- The data collection of this study depends on the public database, and no applicable external data set has been found for external validation.
- The research results have not been further validated by experiments.

## Conclusion

Our study constructed a clinical prognosis prediction model, which was based on gastric cancer and NETs-related lncRNAs. We analyzed the relationship between NETS-related lncRNAs and prognosis, tumor immunity, microenvironment characteristics and treatment of gastric cancer. These results have important guiding value for exploring new treatments for gastric cancer. In the future, we aim to expand our research by collecting additional samples to further validate the reliability and practicality of our model.

### Supplementary Information

Supplementary material 1.

Supplementary material 2.

Supplementary material 3.

Supplementary material 4.

Supplementary material 5.

Supplementary material 6.

Supplementary material 7.

Supplementary material 8.

Supplementary material 9.

Supplementary material 10.

Supplementary material 11.

## Data Availability

The data set involved in this study can be obtained from the TCGA (https://portal.gdc.cancer.gov/). Further inquiries can be directed to the corresponding authors.

## Code availability

All statistical analyses in this study were conducted by R software version 4.3.2.
