# Supplementary material for: Neutrophil extracellular traps-related lncRNAs prognostic signature for gastric cancer and immune infiltration: potential biomarkers for predicting overall survival and clinical therapy
Source: Discov Oncol. 2024 Jul 19;15:291. doi: 10.1007/s12672-024-01164-0 (PMC11264613; doi:10.1007/s12672-024-01164-0)
Supplement: Supplementary file 1 — Supplementary material 1. [file 12672_2024_1164_MOESM1_ESM.docx]

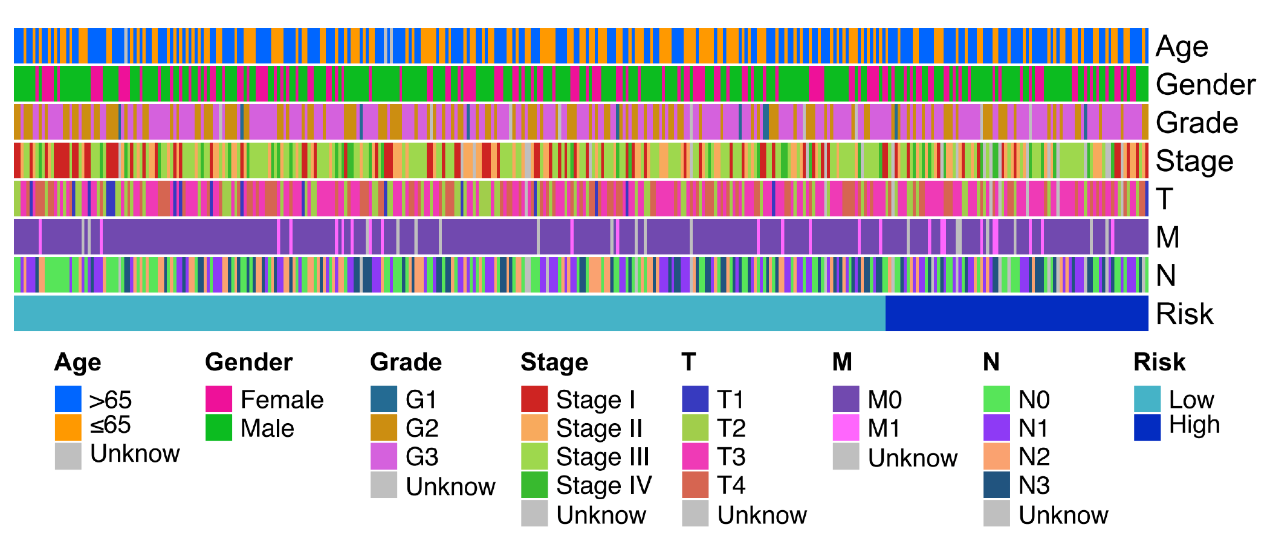


Supplementary figure S1: Clinical heatmap for the differences in clinical characteristics between high and low-risk groups of patients
