# Supplementary material for: Neutrophil extracellular traps-related lncRNAs prognostic signature for gastric cancer and immune infiltration: potential biomarkers for predicting overall survival and clinical therapy
Source: Discov Oncol. 2024 Jul 19;15:291. doi: 10.1007/s12672-024-01164-0 (PMC11264613; doi:10.1007/s12672-024-01164-0)
Supplement: Supplementary file 3 — Supplementary material 3. [file 12672_2024_1164_MOESM3_ESM.docx]

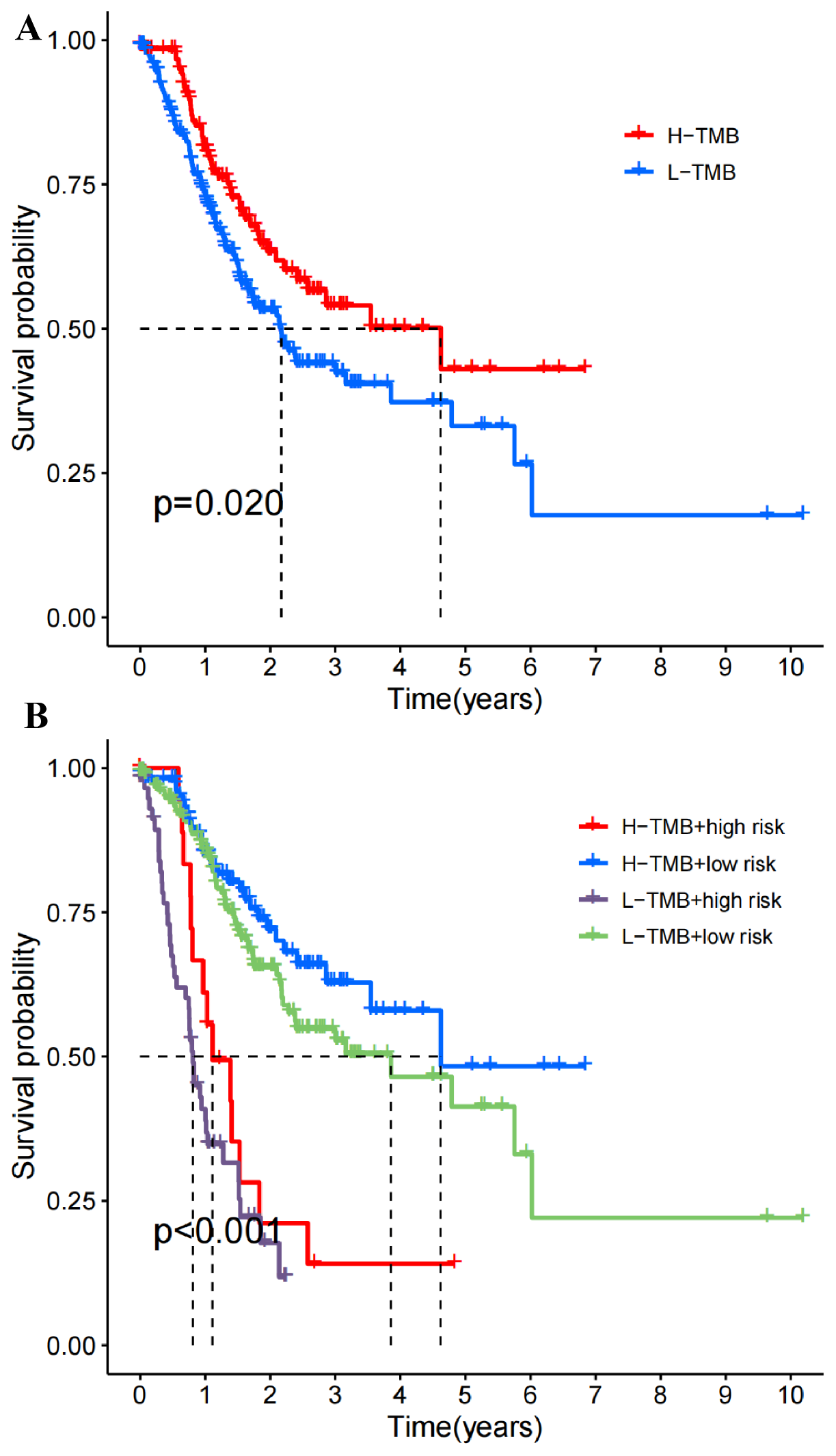


Supplementary Figure S3: (A) K-M survival curve of patients with different mutation States; (B) K-M survival curve of different mutation state patients in high- and low-risk groups.
