# Supplementary material for: Neutrophil extracellular traps-related lncRNAs prognostic signature for gastric cancer and immune infiltration: potential biomarkers for predicting overall survival and clinical therapy
Source: Discov Oncol. 2024 Jul 19;15:291. doi: 10.1007/s12672-024-01164-0 (PMC11264613; doi:10.1007/s12672-024-01164-0)
Supplement: Supplementary file 9 — Supplementary material 9. [file 12672_2024_1164_MOESM9_ESM.docx]

Table assignment

| Factors | Category |  | Assignment |
| --- | --- | --- | --- |
| Gender | Male |  | 1 |
|  | Female |  | 0 |
| Grade | G1 |  | 1 |
|  | G2 |  | 2 |
|  | G3 |  | 3 |
| Stage | 1 |  | 1 |
|  | 2 |  | 2 |
|  | 3 |  | 3 |
|  | 4 |  | 4 |
